# Field‐Controlled Electrical Switch with Liquid Metal

**DOI:** 10.1002/advs.201700169

**Published:** 2017-09-26

**Authors:** James Wissman, Michael D. Dickey, Carmel Majidi

**Affiliations:** ^1^ Mechanical Engineering Carnegie Mellon University Pittsburgh PA 15213 USA; ^2^ Chemical and Biomolecular Engineering NC State University Raleigh NC 27695 USA

**Keywords:** liquid metals, bipolar electrochemistry, bistable, electrical switches, electrowetting

## Abstract

When immersed in an electrolyte, droplets of Ga‐based liquid metal (LM) alloy can be manipulated in ways not possible with conventional electrocapillarity or electrowetting. This study demonstrates how LM electrochemistry can be exploited to coalesce and separate droplets under moderate voltages of ~1–10 V. This novel approach to droplet interaction can be explained with a theory that accounts for oxidation and reduction as well as fluidic instabilities. Based on simulations and experimental analysis, this study finds that droplet separation is governed by a unique limit‐point instability that arises from gradients in bipolar electrochemical reactions that lead to gradients in interfacial tension. The LM coalescence and separation are used to create a field‐programmable electrical switch. As with conventional relays or flip‐flop latch circuits, the system can transition between bistable (separated or coalesced) states, making it useful for memory storage, logic, and shape‐programmable circuitry using entirely liquids instead of solid‐state materials.

## 1 Introduction

Coalescence and separation of liquid droplets are typically governed by fluidic instabilities that arise under static^1^, ^2^ (e.g., liquid bridge separation) or hydrodynamic^3^, ^4^, ^5^, ^6^ (Rayleigh instability) conditions. While much is already known about their role in fluid mechanics (e.g., capillary bridges, continuous jets, and droplet‐to‐droplet impacts), there has been relatively little study of how these instabilities can be harnessed to control droplet interactions in electrochemical systems. Of special interest is the reversible coalescence and separation of liquid droplets through electrowetting or electrochemistry under voltages of ≈1–10 V. Such an ability could enable field‐programmable microfluidics that can be directly operated with conventional microelectronics and power supplies. Moreover, it provides an opportunity to further explore the interplay between interfacial tension, geometry, and fluidic instabilities through spatial control of interfacial energies.

**Figure 1:**
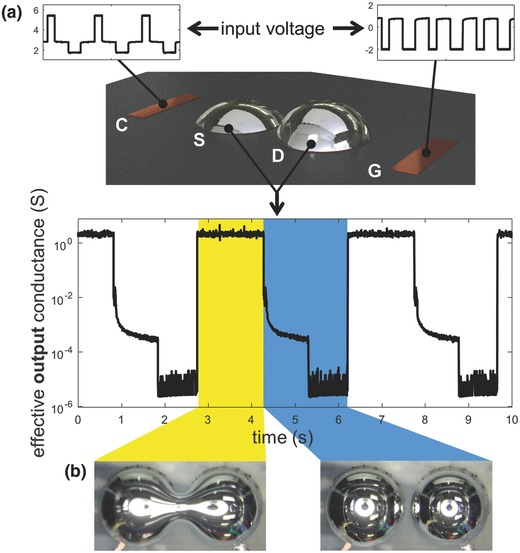
Overview of the “liquid metal transistor.” a) Layout of key electrodes, including the counter (C), the source (S), the drain (D), and the gate (G). The source and drain are wetted with EGaIn. The inset plots refer to the input voltage (relative to the source at 0.85 V) to achieve coalescence and separation. b) Plot of the measured equivalent conductance across the source and drain, which varies by >3 orders of magnitude depending on whether or not drops are coalesced (left) or separate (right).

Several examples of digital microfluidics and liquid‐based switches exist in the literature, though most demand high voltages for conventional electrostatic techniques^7^, ^8^, ^9^, ^10^, ^11^ or activate under outside influences such as environmental corrosion of oxide.^12^ Referring to **Figure** **1**, low‐voltage‐controlled coalescence and separation are accomplished with a pair of liquid metal (LM) droplets immersed in a basic aqueous electrolytic solution. The droplets are anchored to copper pads (referred to as the gate and drain) via alloying. Voltages are applied at these electrodes as well as at two outer copper pads (referred to as the counter and gate) to achieve switching behavior. Like traditional field‐effect transistors, on/off states can be manipulated with the input of electric fields, and a gate–source threshold voltage must be met to achieve off‐to‐on switching (coalescence). In contrast to transistors, this system involves the physical reconfiguration of LM contacts rather than the rearrangement of electrons and holes, and separation requires a fourth (counter) electrode that likewise has gate–source–counter voltage requirements. Conductance between the source and drain changes by over three orders of magnitude depending on whether or not the droplets are coalesced. Further demonstration of switching and details regarding functionality are reported in Video S1 and in the content of the Supporting Information.

**Figure 2:**
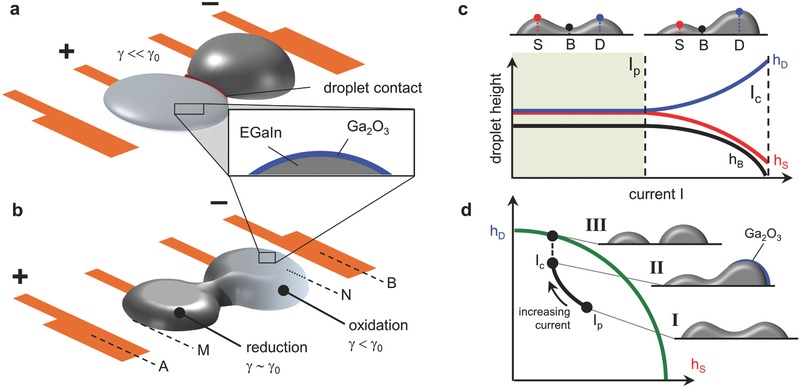
Summary of droplet coalescence and separation behavior. a) An oxidative potential is applied at the source electrode while the gate is negative. This causes spreading of the source EGaIn. Contact and coalescence occur between the source and drain. Positions A, B, M, and N are relevant for Equation (2). b) A positive voltage is applied at the counter relative to the gate. Oxidation occurs on the anodic pole of the EGaIn, and reduction occurs on the cathodic pole, causing a gradient of interfacial tension which eventually makes the system unstable. c) Droplet and bridge height as a function of current when voltage is applied across the counter and gate electrodes. Blue (*h*_D_) is the drain side, red (*h*_S_) is the source side, and black (*h*_B_) is the bridge. d) Heights of LM over the source and drain pads. The green curve follows the heights when drops are separated (limited by volume), and the black curve follows the heights when the drops are coalesced and as current is applied across the outer electrodes.

The LM is a eutectic Ga–In (EGaIn) alloy, which forms a Ga_2_O_3_ surface oxide in aqueous basic environments when placed under an oxidative potential. When such a potential is applied directly to the source electrode (relative to the gate), the associated LM spreads, contacts, and coalescences with the neighboring droplet (**Figure** **2**a). On the other hand, a voltage applied across the gate and counter causes separation under the influence of an oxide‐controlled gradient in interfacial tension (Figure 2b). The latter involves two stages: geometrically constrained droplet deformation during electrochemical oxidation (Figure 2c) followed by capillary bridge separation. This fluidic instability corresponding to a limit‐point in the locus of solutions to the governing Laplace equation (Figure 2d). Such solutions represent the critical point of an energy functional (Π) that accounts for both the interfacial gradient and the incompressibility of the fluid.

This unique approach to controlling liquid droplet interactions builds on new insights in EGaIn electrochemistry and LM–fluid interactions. When immersed in a 1 m NaOH(aq) solution, voltage‐controlled (<10 V) oxidation leads to a dramatic decrease in effective interfacial tension.^13^ Under gravity, the droplet will flatten, which we harness here to bring the droplets closer together and ultimately coalesce. Previously, these and similar low‐voltage electrochemical methods for manipulating LM have been studied for achieving drastic surface area changes,^14^ device reconfiguration,^15^, ^16^ tunable antennas,^17^ and light valving.^18^ While LM droplet coalescence has been studied in water with reductive voltages^14^ and in NaOH solution without applied current (spontaneous coalescence),^19^, ^20^ this work focuses on the controlled use of oxidative potentials to achieve this goal. Furthermore, the method for separation harnesses a novel electrocapillary instability driven by oxide‐induced interfacial tension gradients, which has not before been demonstrated in the literature. In addition to providing experimental evidence and insights into these interface phenomena, we show how such field‐controlled droplet interactions can be used for gated logic. This “liquid metal transistor” (Figure 1) represents the first demonstration of a reversible, bistable fluidic switch that conducts DC electricity and can be operated with low voltage (<10 V). Although not practical as a replacement for solid‐state transistors, it nonetheless demonstrates the ability to create field‐programmable fluidics that are controlled by conventional electrical circuitry.

## 2 LM interfacial tension

The phenomena in Figures 1 and 2 are governed by underlying principles of LM interfacial tension and electrochemistry. In an oxygenated environment, droplets of EGaIn form a self‐passivating Ga_2_O_3_ skin.^21^ When removing the oxide in a bath of NaOH(aq) or HCl(aq), the liquid metal becomes a Newtonian fluid with high interfacial tension (γ* ≈ 0.5 J m^−2^). In this reduced state, a droplet of EGaIn will equilibrate into an energetically stable shape (volume Γ (m^3^)) that minimizes a free energy potential Π (J) subject to geometric constraints. Of special interest here is the case when the droplet wets the surface of a copper electrode through metallic alloying—this alloyed region remains of constant area and interfacial energy. The EGaIn–NaOH solution interface S (m^2^) is then the only surface relevant for calculating potential energy. The equilibrium shape Γ corresponds to a critical point of the energy functional

(1) $$\Pi \;\;=\;\;\oint_{S}\gamma \;dA\;+\;\int_{\Gamma }\left(\rho_{G}-\rho_{S}\right)gz\;dV$$

which accounts for interfacial and gravitational energy while remaining subject to the isoperimetric constraint $\int_{\Gamma }dV\equiv V$. Here, γ is the interfacial tension at the LM–solution interface, ρ_G_ is EGaIn density, ρ_S_ is surrounding solution density, *g* is gravitational acceleration, *z* is the height of a point inside the droplet, and V is the prescribed fluid volume.

The surface oxide is restored when a voltage (Φ) that exceeds the oxidative potential (Φ_O_) is applied across the LM–solution interface. This occurs during coalescence (Figure 2a). Oxide deposition lowers the interfacial tension, which can be roughly approximated by the scaling $\gamma \;\;\approx \;\;\gamma *e^{-\Phi /\Phi_{O}}$. In addition to drastically lowering the tension, surface oxidation increases with greater proximity to the counter electrode due to increased current flow. This results in an interfacial tension gradient and spatial dependency γ = γ(**X**; Φ), where X∈S represents the coordinates of points at the LM–solution interface. Substitution into Equation (1) results in a Dirichlet energy functional that can be minimized using computational techniques. We utilize Surface Evolver,^22^ which uses a gradient descent method to solve this functional and has previously been used to study liquid metal solder.^23^, ^24^, ^25^ For our problem, γ(**X**; Φ) must be input manually since the software does not model voltage gradients or electrochemical interactions.

When brought into contact, EGaIn droplets wetted to two separate electrodes can coalesce and form a stable liquid bridge. For the configuration shown in Figure 2, this requires adequate fluid volume for a given center‐to‐center electrode spacing *s* and pad diameter *D*. To initiate contact, an oxidative potential is applied between one of the droplets and the gate electrode, located opposite the neighboring droplet. This voltage drop causes the oxidizing droplet to preferentially spread toward the gate and thus toward the neighboring EGaIn wetted to the drain pad. Once the two droplets are in nominal contact, they coalesce under the influence of interfacial tension. With the oxidative potential switched off and in the presence of NaOH solution, oxide will be removed and the interfacial tension will increase, though this process can be hastened with a brief (≲1 s) reductive potential applied directly to the metal droplet.

Gradients in interfacial tension can also be induced by applying a current across the EGaIn from two outer electrodes that are not in direct contact with the LM (Figure 2b). Oxidation and reduction occur on the anodic and cathodic poles of the metal, respectively, once a critical end‐to‐end (point M to point N in Figure 2b) voltage drop (ΔΦ_p_) is achieved. Beyond this point, the levels of oxidation and reduction can be tuned by adjusting the applied potential. This phenomenon is referred to as bipolar electrochemistry.^26^, ^27^ It is not limited to liquid metal and has largely been studied with solid metals for creating Janus and striped particles,^28^ generating motion via gas production,^29^ and growing gradients of material.^30^, ^31^ This bipolar redox has been previously observed with GaIn as a growth of gallium oxide on the anodic pole, though it typically behaves as a hindrance to droplet motion^32^, ^33^ and pumping.^34^

Since the experiments were performed in a bath, the voltage drop (ΔΦ) from M to N and the current (*I*) across the outer electrodes are related by the following impedance law

(2) $$\Delta \Phi \;\;=\;\;\frac{I}{2\pi \sigma }\left(\frac{1}{l_{\mathrm{AM}}}-\frac{1}{l_{\mathrm{BM}}}-\frac{1}{l_{\mathrm{AN}}}+\frac{1}{l_{\mathrm{BN}}}\right)$$

This model accounts for Faradaic impedances at the electrodes, caused by mass transport and electron transfer.^35^ Here, σ is the solution conductivity, and *ℓ*_*ij*_ for *i* ∈ *A*, *B* and *j* ∈ *M*, *N* represent the distances between the outer electrodes (A, B) and an intermediate pair of points (M, N), as marked in Figure 2b. This approach is adapted from techniques in geophysics to interpret vertical electrical sounding data^36^, ^37^, ^38^ (also used for measuring resistivity of semiconductor germanium^39^). With Equation (2), one can predict the required current to achieve the necessary potential drop ΔΦ for a specific level of bipolar redox. The use of current also avoids any ambiguities related to the dramatic voltage drops near the electrode interfaces due to Faradaic impedances.

## 3 Results

**Figure 3:**
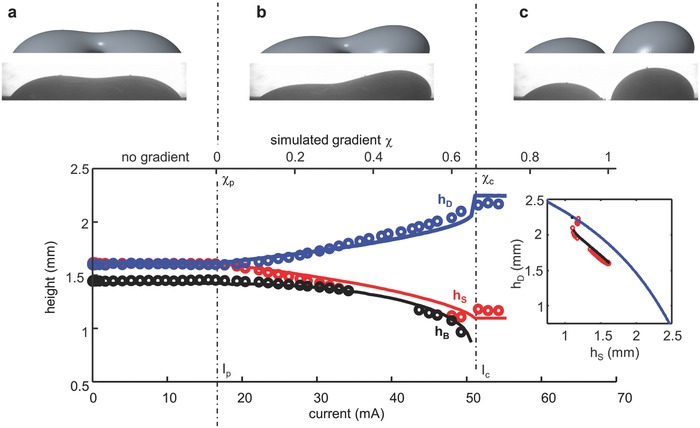
Comparison of Surface Evolver surface tension gradient simulation (plotted as solid lines) to experimental photos (plotted as circular points). a) No gradient. b) Just prior to separation. c) Just after separation.

**Figure 4:**
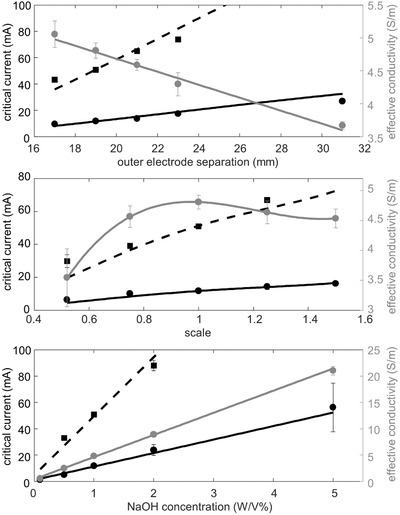
Results concerning droplet separation. Experimental data for movement onset *I*_p_ (circular) and separation *I*_c_ (square) are plotted as points. Theoretical values for movement (solid black) and separation (dashed black) are plotted as lines. The gray points are effective conductivity values fit with a functions (gray lines). Data are reported as a function of outer electrode (gate and counter) separation, overall scale, and NaOH concentration.

Experimental and theoretical results are presented in **Figures** **3** and **4**. The analysis suggests that the kinetics of oxide growth/removal and droplet motion are influenced by geometry (V, *s*, *D*), electrical stimulation (Φ, *I*), and the electrolytic concentration. Following Faraday's electrochemical laws, greater current increases the oxide growth rate while the solution simultaneously etches away the oxide layer. Current can most easily be adjusted by changing the applied potential, although pad geometry and EGaIn volume can also have an impact. Additionally, the NaOH concentration influences the impedance relationship since it dictates the solution conductivity (σ). Understanding these relationships is important for controlling droplet interaction. In the case of coalescence, if Φ is too small, the LM droplet deformation may be insufficient to induce contact. With too much potential, the oxide growth will be excessive, either providing mechanical resistance to droplet deformation or preventing coalescence even after contact. Also, adequate time is required to allow the droplet to spread and make contact. If the spreading period is too long, the liquid metal might make unwanted contact with the outer electrodes or undergo oxide‐mediated fingering.

To separate the droplets, a voltage is applied across the counter and gate electrodes. First, we observe (Figure 3a) that movement does not initiate until a critical current value *I*_p_ (corresponding to ΔΦ_p_). This behavior is reminiscent of electrolysis onset and runs counter to continuous electrowetting (discussed below), which theoretically should have no critical value. We next observe that the coalesced drop shifts toward the grounded gate, which in this case is acting as the cathode (Figure 3b,c). This is, again, in contrast to what is typically seen in continuous electrowetting, during which EGaIn droplets in an NaOH solution move toward the anode.^32^, ^33^ Thus, we conclude that bipolar electrochemistry and oxidation must be the driving factor in our experiments. Oxide growth on the anodic pole (facing the gate/cathode) causes the a dramatic lowering of interfacial tension in the affected area while reduction on the cathodic pole (facing the counter/anode) causes interfacial tension to remain high. To minimize the energy of the system, the liquid metal shifts to lower the surface area of the cathodic pole while the area of the anodic pole grows. Alternatively, this behavior can be explained with the Young–Laplace equation, maintaining a constant change in pressure by increasing the mean curvature where interfacial tension is low and decreasing the curvature where interfacial tension is high. If the interfacial tension gradient is sufficient, it becomes more energetically advantageous to have separate drops, breaking the bridge (at *I*_c_ and ΔΦ_c_).

To further understand the influence of the interfacial tension gradient on a set of coalesced drops, we ran simulations with Surface Evolver. Our simulation begins with droplets coalesced and in an equilibrium configuration, as seen experimentally (also see Video S3 in the Supporting Information). A linear surface energy gradient is then applied, decreasing a normalized surface tension from $\hat{\gamma }=1$ on one side (M) to 1 − χ on the other (N) $\left(\hat{\gamma }=\hat{\gamma }(x,\chi )\right)$. As χ increases, the volume shifts toward the side with lower surface energy. At a critical value χ_c_, the liquid separates into two droplets. As seen in Figure 3, the simulation is qualitatively very similar to what we observe experimentally. The gradient χ represents the constant slope of the imposed surface energy gradient as a function of *x* (distance from end to end). Experimentally, a supplied current *I* (or voltage Φ) produces a particular interfacial tension gradient that, while certainly not linear, can be compared qualitatively to χ. Thus, the critical current *I*_p_ corresponds to χ_p_ = 0 for the onset of bipolar electrochemistry, and the critical value *I*_c_ corresponds to χ_c_ for droplet separation. *I*_p_ is approximated experimentally by observing the onset of droplet motion. *I*_c_ and χ_c_ mark the limit‐point instability of the system in which the bridge formation is unsustainable.

Reasonable agreement between theory and experiment is also demonstrated in Figure 4, which compares predictions from the bipolar electrochemistry model (using Equation (2)) with measurements taken during droplet separation. The plots show the effective conductivity and the electrical current supplied to the bath to initiate droplet motion and to cause separation as a function of outer electrode separation (ℓ_AB_), length scale (scaling pad dimensions, distances, and GaIn volume, but not bath volume), and NaOH concentration. The onset of droplet motion as a metric for bipolar electrolysis onset assumes that movement only occurs when the interfacial tension has been significantly changed by the growth of oxide—an approximation that overestimates current required for bipolar electrolysis since low levels of redox may occur prior to detected motion. Critical values ΔΦ_p_ = 0.165 V and ΔΦ_c_ = 0.72 V were determined experimentally for the reference configuration of outer electrode separation 19 mm, scale 1, and 1% NaOH. These two critical values were used to create curves predicting the movement and break current, respectively.

The distance between the electrodes affects the response of the liquid metal to potential. For example, as the outer electrodes are further separated, the required critical currents for movement and droplet breaking both increase. This is well explained by the theory; the current must flow through a greater length of solution, decreasing the overall electric field strength. Thus, a greater current must be supplied across the counter and gate electrodes to reach the critical ΔΦ. Like outer electrode separation, increasing scale increases distances, thus increasing the required currents for both movement and separation. It was also assumed that bipolar electrolysis and separation occurs at the same ΔΦ_p_ and ΔΦ_c_, regardless of NaOH concentration. (This is particularly oversimplified for ΔΦ_c_ since concentration influences both electrolysis rate and the nonvoltage‐induced reduction rate of gallium oxide, thus influencing the interfacial tension gradient.) As indicated by Equation (2), a decrease in resistivity should result in an equal increase in required current.

## 4 Discussion

The results presented in Figures 3 and 4 show that applied electrical current, geometry, and electrolytic concentration all have an important role in motion and separation of the coalesced EGaIn droplets. In addition to providing validation for the underlying principles related to Equations (1) and (2), the experimental measurements suggest that an “LM transistor” could be tailored to respond to a prescribed electric input. For example, closer outer electrodes and smaller scales result in a lower required current for droplet separation.

The effective conductivities reported in Figure 4 account for boundary effects due to finite bath size. Experiments were performed in baths of dimensions 50 × 75 × 17 mm, but Equation (2) assumes an infinite half space of uniform conductivity. Thus, experimental conductivity measurements (see the Supporting Information) do not account for areas of essentially infinite resistance and underestimate the true value. However, these effective conductivities coupled with Equation (2) more accurately describe the voltage distribution within the bath. Linear fits were applied to the outer electrode separation and NaOH concentration conductivity data, while a cubic polynomial was fitted to the scale conductivity data (see the Supporting Information).

There is also a key difference between the two critical voltage drops ΔΦ_p_ and ΔΦ_c_. ΔΦ_p_ is geometry invariant in the sense that, regardless of outer electrode separation or scale, it should always mark the onset of bipolar electrolysis. The voltage distribution between the endpoints does not influence the fact that redox occurs. On the other hand, ΔΦ_c_ is a less accurate approximation because separation is dependent on the voltage distribution between the endpoints. The same ΔΦ_c_ may be reached for multiple geometries, but the voltage distribution will differ for each, resulting in differing areas of oxidation and reduction, and differing interfacial tension gradients. In other words, separation occurs when an adequate interfacial tension gradient is achieved, and ΔΦ_c_ provides an approximation for when this gradient is reached.

Experimental deviation from the theory was minor and generally explicable. Limitations in our testing circuit (<10 V and <100 mA) prevented droplet breaking at the pad distances greater than 23 mm. Moreover, it is still clear from the top plot in Figure 4 that the theory (which uses critical voltage drops tailored for a separation of 19 mm) diverges from experimental values at larger electrode separations. Rate effects could be the cause, although experiments were designed to be quasistatic (voltage increased at 0.1 V s^−1^). Alternatively, deviation could be due to the interference of bubbles and turbulence (electrolysis or Marangoni flow induced) at close proximity to the electrodes. Geometry could thus influence behavior in ways that are not captured by the basic bipolar electrochemistry formula. We speculate that the geometric influences which caused deviation in the electrode separation are nearly proportionate with dimension, allowing the theory to predict the behavior better with scale. However, separation does not occur reliably at small scales (0.5×) or large scales(≥1.25×). At smaller scales, the primary reason is interference of bubbles that block current flow. At larger scales, the reasoning is less clear, though an upper limit for separation current appears to be the cause (see the Supporting Information). Separation also fails to occur at low NaOH concentrations (1%), where the ions were insufficient to reach the required interfacial tension gradient. At higher concentrations (5%), separation would occur at currents beyond the range of our testing circuit.

It should be noted that an alternative mechanism for inducing gradients in interfacial tension is through electrocapillarity, which follows the Young–Lippmann equation for relating γ and Φ. This effect has been used to cause fluid motion through the so‐called continuous electrowetting and the Marangoni effect.^34^ Assuming that the LM droplet is equipotential (due to its high conductivity), there exists a variation of voltage across the drop due to the relatively low conductivity of the surrounding solution. As suggested by the Young–Lippmann equation, a gradient in interfacial tension develops along the liquid metal, resulting in a force that can move either the droplet or the surrounding electrolytic solution. Originally, this phenomenon was applied to manipulate mercury slugs.^40^, ^41^ More recently, it has been examined for EGaIn^32^, ^33^ and applied to microfluidic pumping^34^ and mixing.^42^ For gradients induced by electrocapillarity, EGaIn droplets immersed in NaOH(aq) move toward the anode (positively charged electrode) and the surrounding fluid is pushed in the opposite direction.^32^, ^33^ While also of general interest, electrocapillarity does not achieve the same dramatic interfacial tension change as oxide growth, which can reach interfacial energies of nearly 0 J m^−2^, as discussed in the literature.^43^ Further, as discussed above, our experiments indicate that although it occurs simultaneously with bipolar electrolysis, electrocapillarity is not the driving mechanism in this work.

## 5 Summary and Outlook

We present a fluidic electrical switch that reversibly changes its electrical conductivity by three orders of magnitude in response to moderate applied voltage (1–10 V). This “liquid transistor” is the first soft‐matter electrical switch that operates with voltages similar to that of conventional solid‐state transistors. LM droplet separation is controlled by a novel fluidic instability that is driven by a field‐controlled gradient in interfacial tension and has not before observed in fluidic electrowetting or LM droplet manipulation. Experimental measurements are in good agreement with theoretical predictions based on fluid mechanics and bipolar electrochemistry. In addition to explaining the observed electrocapillary behavior, the theory can inform the design of physically reconfigurable liquid metal electronics. Potential applications include field‐programmable gate arrays, reconfigurable antennas, and nonvolatile memory storage devices that are mechanically soft and highly deformable. Such advancements could accelerate further progress in the emerging field of LM‐based soft microfluidic electronics.

We have demonstrated the controlled coalescence and separation of anchored LM droplets with the application of electric fields and explained the phenomena. Dramatic decreases in LM interfacial tension under direct oxidation enable droplet contact and coalescence. Separation, however, is driven by bipolar electrochemical interactions that induce an oxide gradient and manipulate the interfacial energy between the LM and the electrolytic solution, leading to instabilities. Potential applications of this bistable response include soft‐matter switches, reconfigurable electronics, and analogs of solid‐state circuits in liquid environments. The work presented here primarily focuses on quasistatic behavior, where the limit‐point instability is governed by interfacial tension. However, rapid pulses of current introduce inertial effects. In principle, cyclic voltage inputs could be used at the natural frequency of the coalesced drops to further decrease separation voltage and to avoid bubbling at the gate and counter electrodes. Furthermore, typical fluidic phenomena such as Rayleigh instabilities could be leveraged to achieve shape programmability within LM circuits.

The onset of LM motion and trends for droplet separation can be predicted with theories from bipolar electrochemistry. Although particularly useful for informing switch design and establishing a general understanding of electrocapillary behavior, more can be learned on the behavior of LM under the influence of applied potentials. Particularly, the proximity of the outer electrodes to the LM droplets appears to have an impact on the bipolar electrochemistry, which is not captured by the theory reported in this paper. Furthermore, models capturing the dynamics of the electrolytic solution (with Marangoni flows and bubbles due to electrolysis) and their interaction with the geometry could produce new and further optimized designs for reconfigurable circuits. A fully predictive Surface Evolver simulation could be designed with precise relationships between current input, reaction rates (oxidation and reduction, both electrical and solution‐induced), and effective interfacial tension. This is, however, beyond the scope of the work presented here, which instead shows predictions for a variety of designs based on a single set of experimentally gathered critical values (ΔΦ_p_ and ΔΦ_c_). Also, as demonstrated in this work, the critical voltage values work well across a wide range of design changes, but there are limitations to the value for separation. In particular, if we change the shape of the LM body (change LM volume, source/drain separation, source/drain pad shape, etc.), the critical voltage for separation would be expected to vary due to changing requirements in interfacial tension gradients (e.g., separation may never occur if LM volume is too large).

One area that requires further study is device lifetime. Although the current system is limited by the corrosion of the copper electrodes during oxidation, this could be remedied with more inert electrodes, such as gold or graphite. The lifetime would then likely be limited by chemical interactions of gallium. In particular, NaOH slowly converts gallium to gallates, [Ga(OH)_4_]^−^, eventually causing the liquid metal to lose its eutectic/near‐eutectic point.^34^ HCl solution is also commonly used with gallium–indium, but would likewise slowly produce gallium chloride.^44^ Given this information, further investigation into alternative solutions is warranted.

Neutral pH electrolyte baths also present interesting possibilities. In the work presented here, basic NaOH solution was used because the dramatic spreading (particularly for coalescence) is not seen in neutral baths. Instead, oxide rapidly grows too thick, indicating that the competition between electrochemical oxidation and oxide removal through bath chemistry is required.^13^ However, if coalescence could be achieved in a neutral bath, the shape would be held by the ever‐present oxide layer even at subcritical volumes of LM. With a subcritical volume, merely reducing the drops would cause separation as surface area is essentially minimized. In this case, no bipolar electrochemistry or interfacial tension gradient would be required for separation. The above improvements, along with bubble‐reducing techniques such specialized electrodes,^45^, ^46^ can improve feasibility of these reconfigurable LM microfluidics. It may also be interesting to explore the use of alternating current for mitigating the formation of bubbles. While promising for some microfluidic systems,^47^ it has been observed that in the case of EGaIn, greater voltage is required to remove oxide at higher AC frequencies.^48^ Channels could also be used to manipulate the electric field strength. Droplets placed in a narrow channel could increase the required voltage bias due to the higher electrical resistance. Higher resistance can also be achieved with lower NaOH percents at the cost of higher voltage requirements, slower oxide growth, and slower overall behavior of the system. These trade‐offs reflect the importance of furthering our understanding of these systems in order to optimize designs for varying applications in soft‐matter electronics and shape‐programmable media.

## Conflict of Interest

The authors declare no conflict of interest.

## Supporting information

Supplementary

Supplementary

Supplementary

Supplementary

Supplementary
