# Supplementary material for: Field‐Controlled Electrical Switch with Liquid Metal
Source: Adv Sci (Weinh). 2017 Sep 26;4(12):1700169. doi: 10.1002/advs.201700169 (PMC5737232; doi:10.1002/advs.201700169)
Supplement: Supplementary file 1 — Supplementary [file ADVS-4-na-s001.pdf]

Copyright WILEY-VCH Verlag GmbH & Co. KGaA, 69469 Weinheim, Germany, 2017.

ADVANCED SCIENCE
Open Access

Supporting Information

for *Adv. Sci.,* DOI: 10.1002/advs.201700169

Field-Controlled Electrical Switch with Liquid Metal

*James Wissman, Michael D. Dickey, and Carmel Majidi**

Field-controlled electrical switch with liquid metal – supplementary information

James Wissman,[1] Michael D. Dickey,[2] and Carmel Majidi[1]

1) Mechanical Engineering, Carnegie Mellon University, Pittsburgh, PA 15213, USA

2) Chemical & Biomolecular Engineering, NC State University, Raleigh, NC 27695, USA

A. Sample Layout and Fabrication

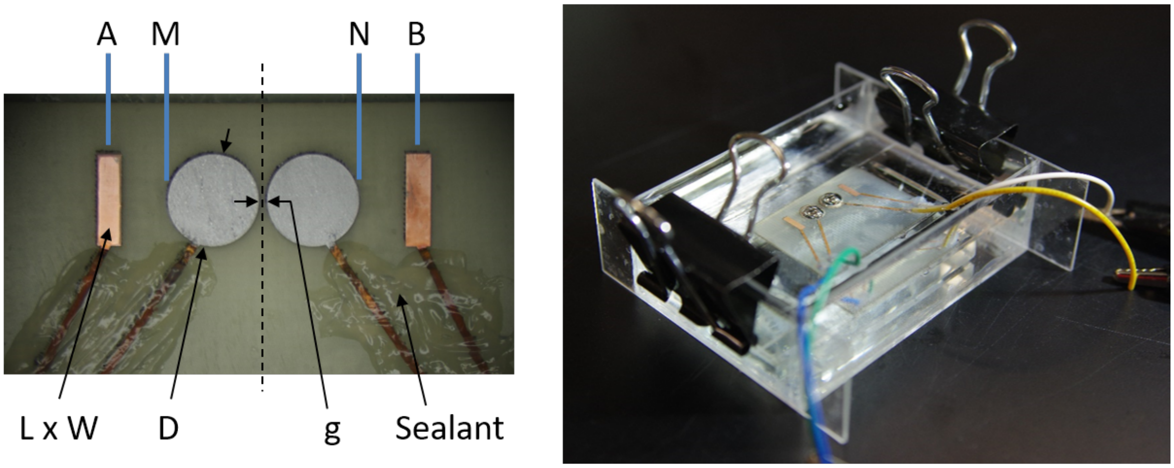

Supplementary Figure 1 – Sample layout and general testing bath. (a) Electrode orientation and dimensions. (b) Testing bath.

Test samples were fabricated on standard copper clad board (0.5 oz. FR4) using a commercial UV laser patterning system (LPKF, ProtoLaser U3). As seen in Supplementary Figure 1, samples are symmetric and consist of two outer rectangular pads of dimensions L x W and two inner circular pads of diameter D. Note that the two circular pads are silver/gray due to alloying with GaIn. Conductive pathways leading away from the electrodes were insulated with Sil-Poxy (Smooth-On) or Loctite Quick Set Epoxy. Standard wiring was soldered to copper contact pads (also insulated with adhesive) for interfacing with external electronics. The base design comprised the following dimensions: D = 5.642 mm (pad surface area of 25 mm^2), W = 1.5 mm, L = 5.642 mm, G = 0.5 mm, and length from A to B l_{AB} = 19 mm. Two droplets of GaIn from an 18G dispensing needle and one drop from a 25G needle, amounting to a volume of approximately 51 mm^3 (droplet volume methods are described below), were used for the base design. Parametric testing consisted of varying only l_{AB} (outer electrode separation) and scaling the entire device (including GaIn volume) while keeping the NaOH weight to volume concentration constant at 1%. When testing for NaOH concentration effects, the base dimensions were used.

Testing baths were constructed of four 25 x 75 mm and one 50 x 75 mm glass slides. Sil-Poxy or Loctite Epoxy was used to adhere and seal the edges, forming a box (Supplementary Figure 1) suitable for taking side profile videos and images without distortion. The FR4 samples were adhered to 50 x 75 glass slides, allowing a snug fit in the baths, preventing twisting. A 2′′ x 1 ′′ x 0.25 ′′ streak plate (United Scientific) was used as a spacer between the bottom of the bath and the sample (preventing poor alignment due to sealant along the edges of the box). Binder clips were used to pin the sample in place. To create the appropriate solution concentration of sodium hydroxide, NaOH pellets (BDH9292, VWR) were added to deionized water (3190K731 , McMaster-Carr), mixed, and allowed to dissolve.

B. GaIn Volume Creation and Measurement

Precise volumes of GaIn were produced by quasi-statically dispensing droplets into a bath of 1% W/V NaOH solution (Supplementary Figure 2). This was performed with a Harvard Apparatus syringe pump (PHD 2000) and dispensing needles (C-U Innovations) of various gauge, ranging from 14G to 27G. While varying the flowrate with a single needle produces a range of droplet volumes, we found that quasi-static production was more repeatable. The volume of an individual drop is determined by the competition between gravitational and surface tension forces. The instability point, when the droplet falls, can be approximated with $2\pi R\gamma = (\rho_G - \rho_S)Vg$, where γ is the GaIn/solution interfacial tension, R is the inner radius of the syringe, ρ_G is the density of GaIn, ρ_S is the density of the solution, V is the volume of the GaIn droplet, and g is gravitational acceleration. This equation is referred to as Tate's law and is commonly used to determine surface tension values [1, 2, 3].

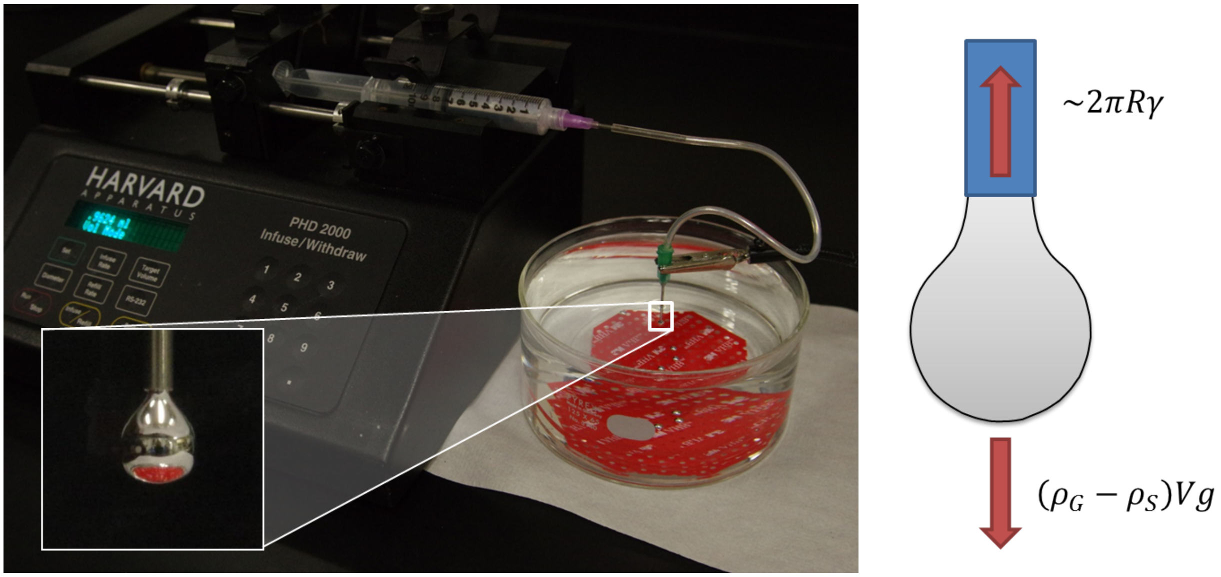

Supplementary Figure 2 – Left: Setup for creating repeatable GaIn volumes. Left inset: Profile of GaIn droplet deposited from a deposition needle. Right: Schematic laying out the forces governing droplet volume.

After creating repeatable droplets, the actual volume had to be determined. Images were taken with an optical stereoscope to extract values for droplet diameter. Assuming spherical droplets was insufficient due to the substantial volume and density. Instead, experimental measurements were compared to simulated results from Surface Evolver, taking γ as 500 mJ/m^2, ρ_G as 6.25 g/cm^3, and ρ_S as 1 g/cm^3. The comparison and results from trials with varying needle diameter are reported in Supplementary Figure 3. The theory described above generally overestimates the experimental results by about 20%, likely due the approximation that separation occurs at the tip of the needle. Several groups have applied corrections to Tate's law [2, 3], increasing prediction accuracy, but none were used for this study since measured values were sufficient. In most cases, desired volumes of liquid metal were produced by combining two or more droplets from a single or multiple needles.

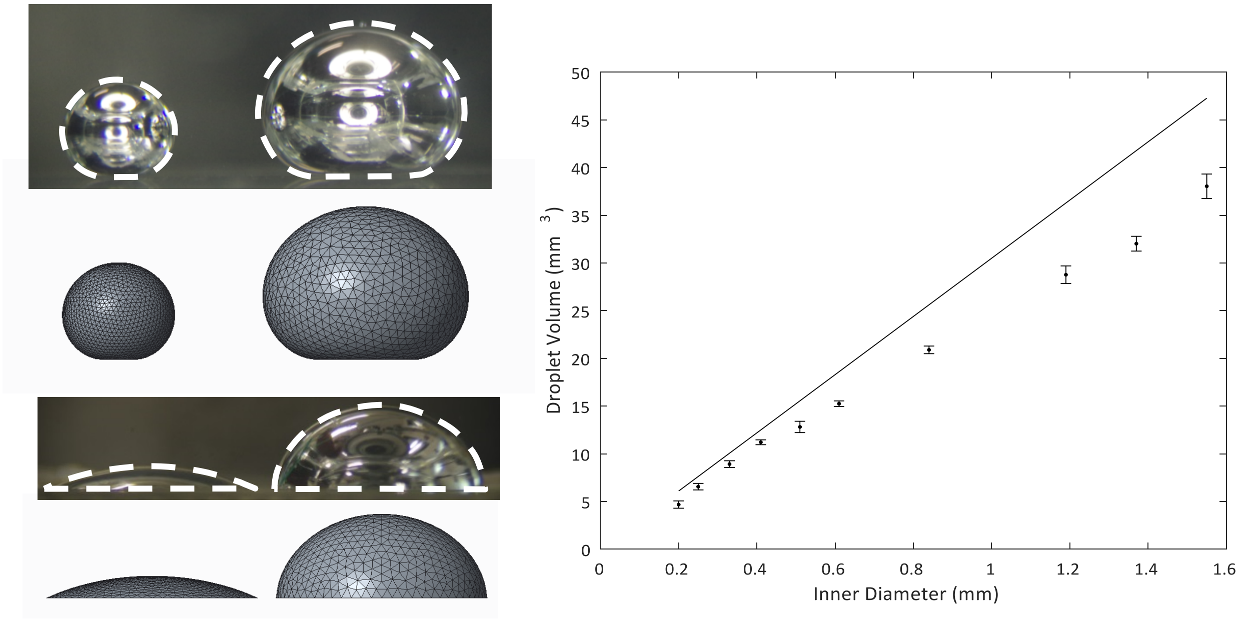

Supplementary Figure 3 – Left: Comparison of experimental images to Surface Evolver results. The small droplet is approximately 7.3 mm^3 and the large droplet is 39.8 mm^3, according to Surface Evolver. The small cap and large cap are 7.8 mm^3 and 37.8 mm^3, respectively. Right: Experimental results (points) for creating droplet volumes based on needle inner diameter (ID) compared to simplified theory based on weight and surface tension (line). Flowrates: 0.2 mm ID – 100 µL/min, 0.25 to 0.41 mm ID – 250 µL/min, 0.51 to 0.84 mm ID – 500 µL/min, 1.19 to 1.55 mm ID – 1000 µL/min.

C. Droplet Control and Electrical Monitoring

For the characterization experiments, an Arduino UNO R3 microcontroller was used in conjunction with three shields (see Supplementary Figure 4). Voltage was supplied by a dual DC power supply (Hewlett Packard) and controlled (V_{in}) with a Power Digital to Analog Converter (DAC) Shield (Visgence, Inc.). Second, an Extended Analog to Digital Converter (ADC) Shield (Mayhew Labs) was used to measure voltages (A#). Lastly, a custom shield was designed and fabricated to provide additional control and signal conditioning. The custom shield contains 3 solid state relays (SSR#) (CS128, Coto Technology) for directing voltage application to 3 of the 4 electrodes (Counter C, Source S, Drain D, and Gate G) associated with the liquid metal switch (the gate is always grounded). To acquire data on current, the voltage across a 1 ohm shunt resistor (R4) was amplified by an instrumentation amplifier (IA) (AD623BRZ-R7, Analog Devices Inc.). The associated gain could be adjusted during testing with a digital rheostat (AD5270BRMZ-100, Analog Devices Inc.). The DAC, ADC, and rheostat were all controlled using serial peripheral interface (SPI) communication through the Arduino. Simple digital signals (d#) from the Arduino controlled the chip (LTC1859, Linear Technology) associated with the Extended ADC Shield allowed for 16-bit resolution and a range of ±10V but was limited by an internal input resistance of 42kΩ for unipolar measurements and 31kΩ for bipolar. As a result, a quad operation amplifier (op amp) buffer (AD8244BRMZ, Analog Devices Inc.) was added to the custom shield to produce low impedance outputs, thus avoiding measurement inaccuracies due to voltage divider effects within the ADC. The entire system was controlled with a custom MATLAB graphical user interface.

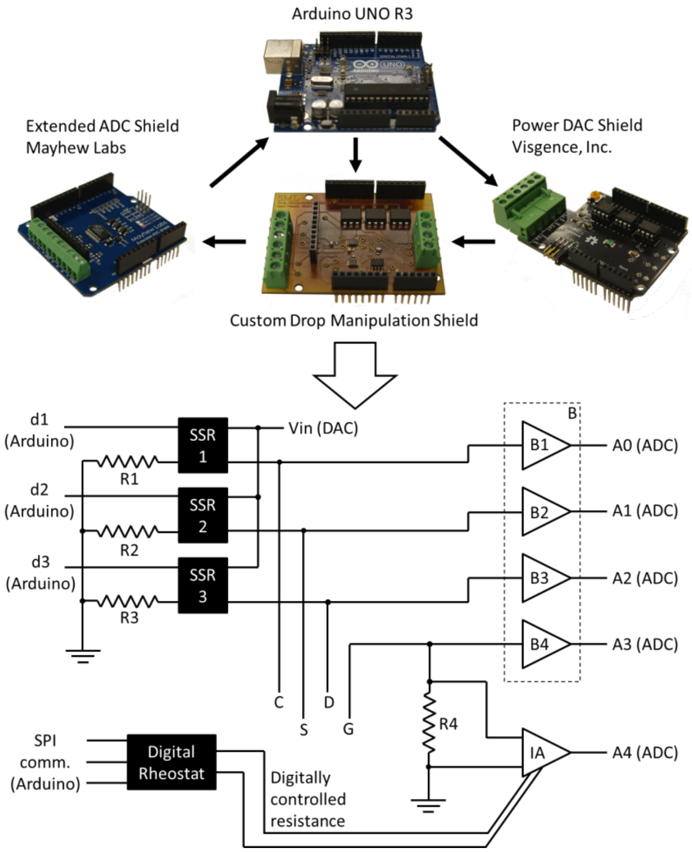

Supplementary Figure 4 – Top: Photographs of the Arduino and three shields. Bottom: Simplified circuit diagram of the custom shield. SSR – Solid state relay. B – Quad op amp buffer. IA – Instrumentation amplifier. d# – Digital input from Arduino. A# – Voltage to be measured. R1-3 – 330Ω current limiting resistors. R4 – 1Ω shunt resistor. C – Counter electrode. S – Source electrode. D – Drain electrode. G – Gate electrode.

Voltage was first applied to the source electrode (~2.5V) to induce spreading and coalescence (while the gate was always grounded). In most cases, a brief (~1 sec) reductive voltage (~-3V) was applied to the source electrode to hasten the removal of oxide immediately after coalescence. This was especially useful for low NaOH concentrations (0.1% or 0.5%) but was detrimental to successful coalescence at higher concentrations (5%) as the snap back motion was rapid enough to cause separation. Next, the voltage applied to the counter was increased in approximately 0.1V increments every second until the droplets separated or until the upper limit of the equipment (~8.5V for ±10V input to the DAC) was reached. After, the voltage applied to the counter was reduced to 0V. The voltages applied at all 4 electrodes and the supplied currents were recorded at 250Hz.

An example of time versus voltage and time versus current is shown in Supplementary Figure 5. The voltage of the source and drain are initially below 0V due to the difference in electrode potential between EGaIn and copper. Oxidation for coalescence and reduction for oxide removal correspond to the first spikes in the plots. Afterwards, the counter electrode increases steadily in voltage. At approximately 2.1V, electrolysis begins across the counter and gate. Next, droplet movement initiates, though there is clear indication in the electrical data. Instead, data from profile videos must be used to determine motion timestamps. While coalesced, the source and drain are approximately equipotential.

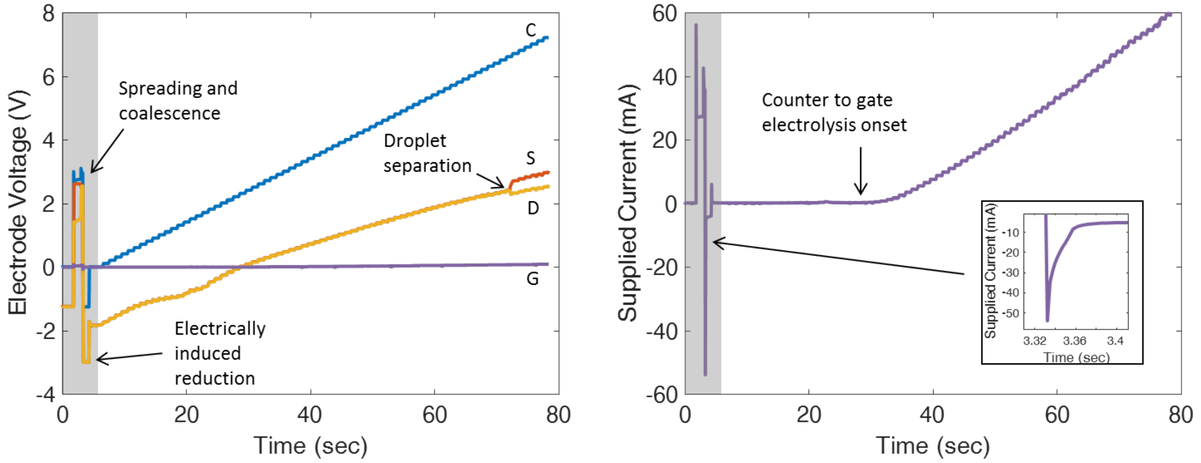

Supplementary Figure 5 – Raw voltage and current readings. The gray areas indicate times when spreading and brief reduction is occurring for the coalescence process. Left: Voltage as a function of time for the counter (C), source (S), drain (D), and gate (G). Right: Current as a function of time for the system. The inset is a zoomed-in plot for a current spike.

After separation at a counter electrode voltage of 6.6V and a supplied current of 50.9 mA, the source and drain voltage diverge due to the significant NaOH resistance now separating them. This feature was used to automate the detection of droplet separation.

Current across the 1 ohm shunt resistor is also plotted in Supplementary Figure 5. From this data, the onset of electrolysis across the counter and gate electrodes can be detected. This data is correlated with voltage data and video data to extract critical currents for movement and separation. There are also spikes and approximately exponential decays in current that can be identified, indicating the presence of capacitive effects. Since these spikes were not observed when replacing the sample/NaOH bath with resistors, it can be concluded that the capacitance is a result of the formation of a double layer at the surface of the liquid metal. Changing potential rapidly causes a current spike as the double layer capacitor discharges.

D. Visual Droplet Monitoring and Image Analysis

Visual data was required for determining the onset of droplet deformation and to quantify the system behavior. Top-down videos provided information on bridge width, but profile videos proved to be more informative by more clearly indicating the shift of mass during droplet separation. Profile videos of droplet deformation were recorded through a stereo microscope (Carton SPZT 50) with a Pentax K3 digital SLR. The overall setup is shown in Supplementary Figure 6a. To acquire a profile view of the liquid metal, a 1st surface mirror was placed at approximately 45°, and the sample was backlit to increase contrast. The experiment could then be magnified and viewed live on a computer monitor through an HDMI connection. In most cases, 4 videos were taken for every 10 tests – on the 1st, 2nd, 5th, and 10th.

To quantify data from video recordings, frames (15 per second) were systematically processed (Supplementary Figure 6b-f) using MATLAB's Image Processing Toolbox. First frames were extracted, cropped, and straightened. The images were then converted to greyscale (MATLAB: rgb2gray), followed by a conversion to black and white with a specified luminance threshold. Irrelevant objects such as bubbles separated from the main body were removed using a filter for connected pixels (MATLAB:

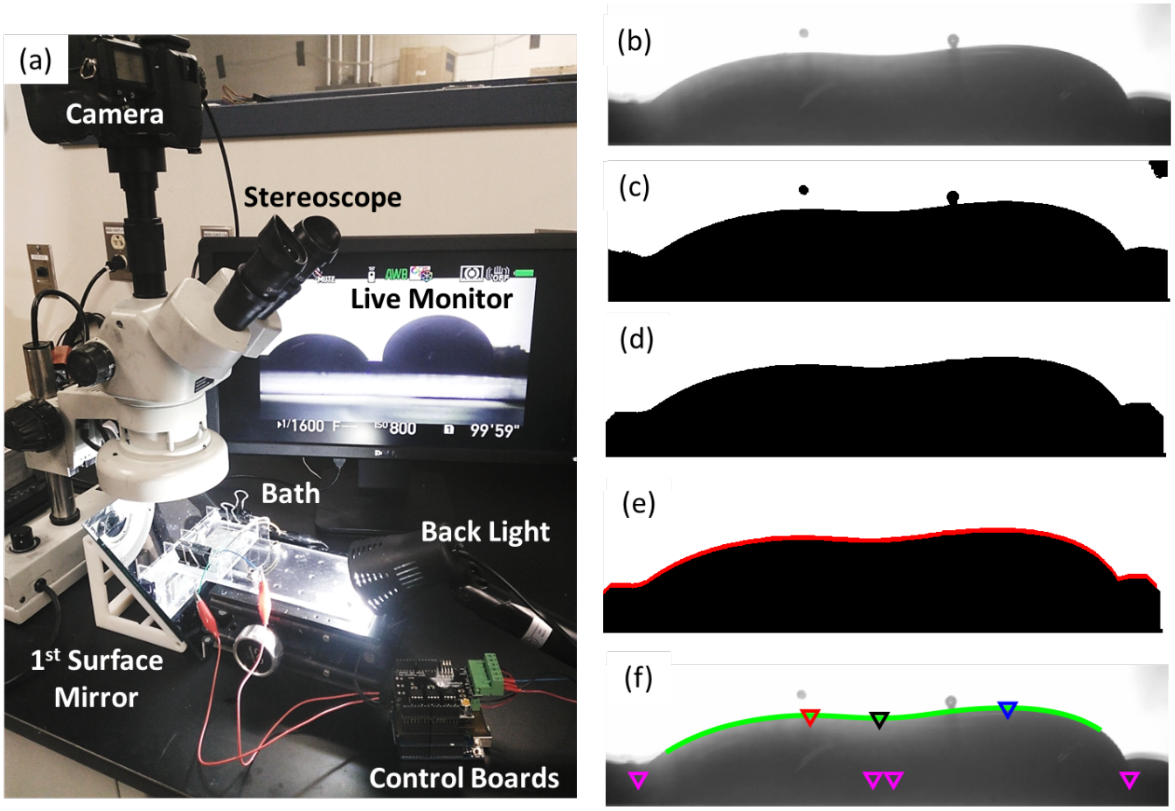

Supplementary Figure 6 – (a) Experimental setup. (b) Cropping and straightening of video. (c) Thresholding to acquire a black and white image. (d) Removal of bubbles and extraneous pixels with morphological functions. (e) Identification of top surface in red. (f) Fitting of polynomial curve and identification of min and max points. Pad edges are marked with magenta.

bwareaopen), and bubbles attached to the surface of the liquid metal could be removed using morphological dilation followed by erosion (combined into one MATLAB function: imclose). The top surface of the droplet could then be identified and points could be extracted. Finally, a polynomial curve was fitted (MATLAB: polyfit) to the extracted points to smooth any roughness due to pixilation and to facilitate the extraction of minimum and maximum liquid metal height values.

A number of approaches were taken to determine the onset of droplet motion. First, the motion of points of interest (such maximum height locations) along the profile could be tracked. Plots such as those shown in Figure 3 of the main document could be used to approximate when and at what current input the liquid metal begins to shift. Alternatively, the polynomial profile curve can be compared to a reference. The profile under zero deformation works as a useful reference (green line in lower image of Supplementary Figure 7) for most comparisons. The deformed profile (blue line in lower image of Supplementary Figure 7) can then be compared to the reference using root mean square (RMS) distance, coefficient of determination R^2, or Hausdorff distance [4]. Tracking the area of a particular section of the profile (such as that shaded in red in Supplementary Figure 7) can also provide information for determining timestamps coinciding with movement. However, deviations due to bubbles and changing light conditions caused noise that made it difficult to locate small deformations, particularly impacting samples with slower responses (large outer electrode separation, low NaOH concentration, etc.). As a result, the most reliable and repeatable method was visual inspection of the videos or video frames. Syncing camera data with voltage/current data from the electronic setup allowed for the determination of current

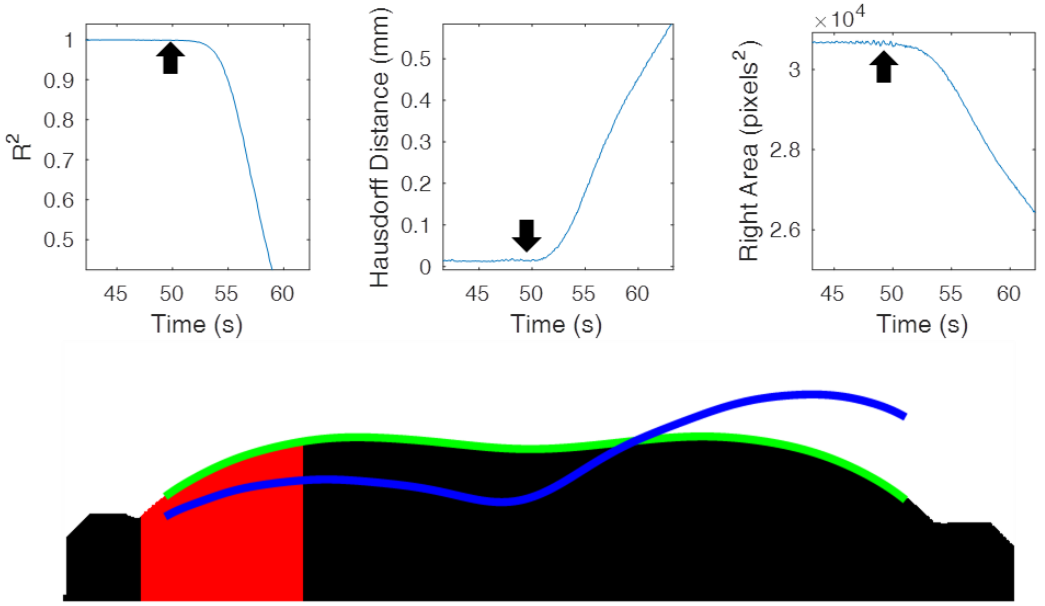

Supplementary Figure 7 – Methods for determining droplet motion. The arrows in the plots approximate where motion begins. (Top left) – R squared value for comparing the non-deformed (green line) and deformed (blue line) shapes. (Top middle) – Hausdorff distance associated with the same two curves. (Top right) – Change in pixel area, indicated by the area shaded in red.

E. NaOH Conductivity

Conductivity of a material is usually determined using Ohm's law. However, the electrochemical interface of bulk solution and electrodes complicates the system [5, 6]. A single electrochemical cell, consisting of solution and two electrodes, is approximated as a circuit in Supplementary Figure 8a. At each interface, an electric double layer forms which behaves like a capacitor (C). A Faradaic impedance, involving mass and electron transport, is represented as a component (Z) in parallel. It should be noted that while the bulk resistance R follows Ohm's law, the Faradaic impedance does not. Finally, the electrode potential is represented with ΔU. To calculate the solution conductivity, the resistance R is required, but the interface effects interfere. In many cases, conductivity measurements are taken by applying alternating currents and simplifying the circuit to a single capacitor and a single resistor in series. However, given that all experiments were performed with direct current, a direct current approach [6] was taken, as seen in Supplementary Figure 8b. If the electrodes and probes are all the same material, the equivalent circuit can be simplified to Supplementary Figure 8c. The electrode potentials then cancel each other out, simplifying the problem. The resistance R_2 is then a function of the current I and the potential difference $\Delta\Phi$, which can be determined directly with the two probes. The conductivity is then a function of the measured resistance and dimensions of the solution and electrodes.

Theoretical modeling with the given geometry was non-trivial due to the fact that a bath and co-planar electrodes were used rather than a simple tube filled with solution and 4 copper electrodes. With this in mind, equations from vertical electrical sounding (VES) were used, as described in the main document. Equation (2) can be rearranged to solve for the conductivity. Test electrodes (examples shown in Supplementary Figure 9) were used to gather the necessary data. All samples were created with the

same FR4 board, laser patterning techniques, and sealing/insulating methods as described previously. The most general design (Supplementary Figure 9a) was created to match the dimensions used for the parametric testing of liquid metal bipolar electrochemistry. However, instead of having a source and drain pad, copper pads were placed where the outer edges of liquid metal would be located. As with the bipolar electrochemistry testing, these sample dimensions were adjusted, scaled, and tested in various NaOH concentrations. Typical testing procedure involved increasing the voltage in steps of 0.1 V/sec across the outer electrodes while recording current and the voltage between the two inner probes. Plotting the difference voltage difference between the probes versus current reveals a linear increase, starting from 0V at 0 mA. This information is then plugged into equation (2) along with dimensions to determine conductivity.

In bipolar electrochemistry, it is generally assumed (though often ignored) that the bipolar electrode (liquid metal in this case) will influence the electric field [7]. Specifically, the high conductivity of the electrode results in a decrease in electric field strength across its physical area. This is a result of the high conductivity and near-equipotential of the electrode. In order to test this, the designs in Supplementary Figure 9b and c were implemented to measure the potential difference in the same relative locations as Supplementary Figure 9a (the outer edge of liquid metal). In Supplementary Figure 9c, liquid metal is included. Supplementary Figure 9b is identical except for the lack of liquid metal. Any large influence of the bipolar electrode should appear as a difference in measured conductivity. However, as shown by the plot in Supplementary Figure 9, there was no significant difference. Given this information, the influence of the liquid metal conductivity was not considered during theoretical calculations. If a narrow tube were used instead of a bath, however, the liquid metal (then taking up a sizeable cross-sectional area) could have a far larger impact and would possibly have to be included for theoretical accuracy.

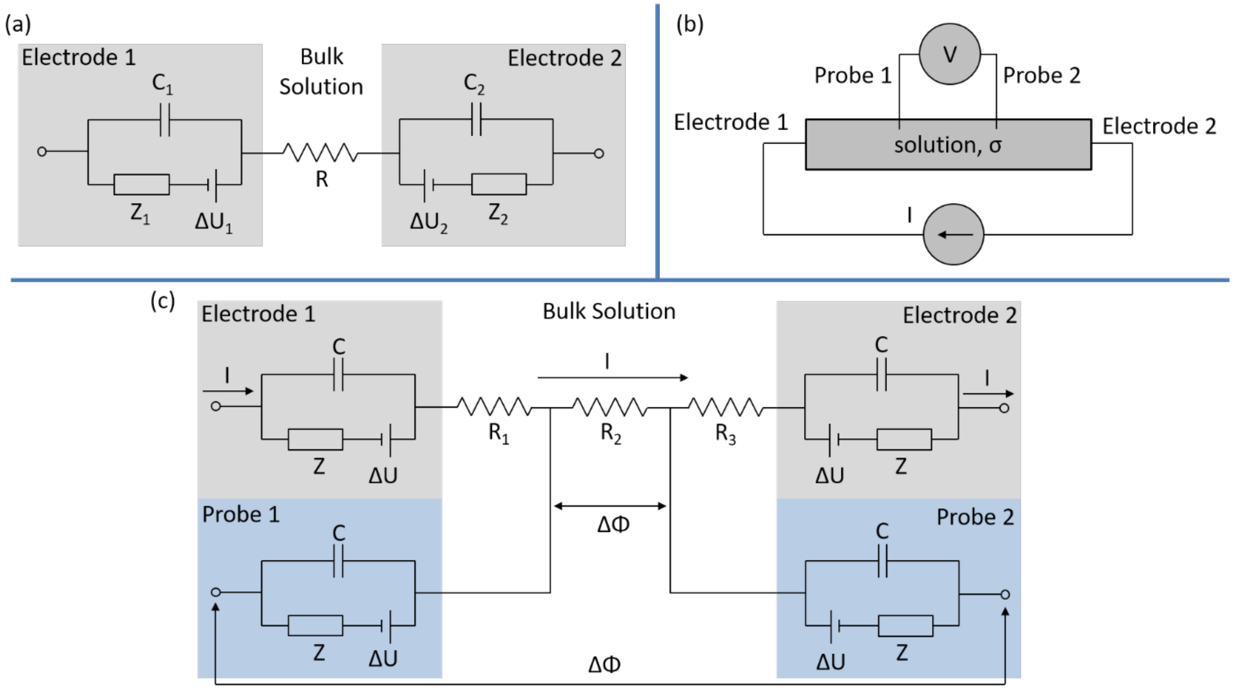

Supplementary Figure 8 – Electrochemical circuitry. (a) A simple electrode-electrode-electrolyte system. (b) The general approach for determining solution conductivity using direct current. (c) The circuit equivalent of (b).

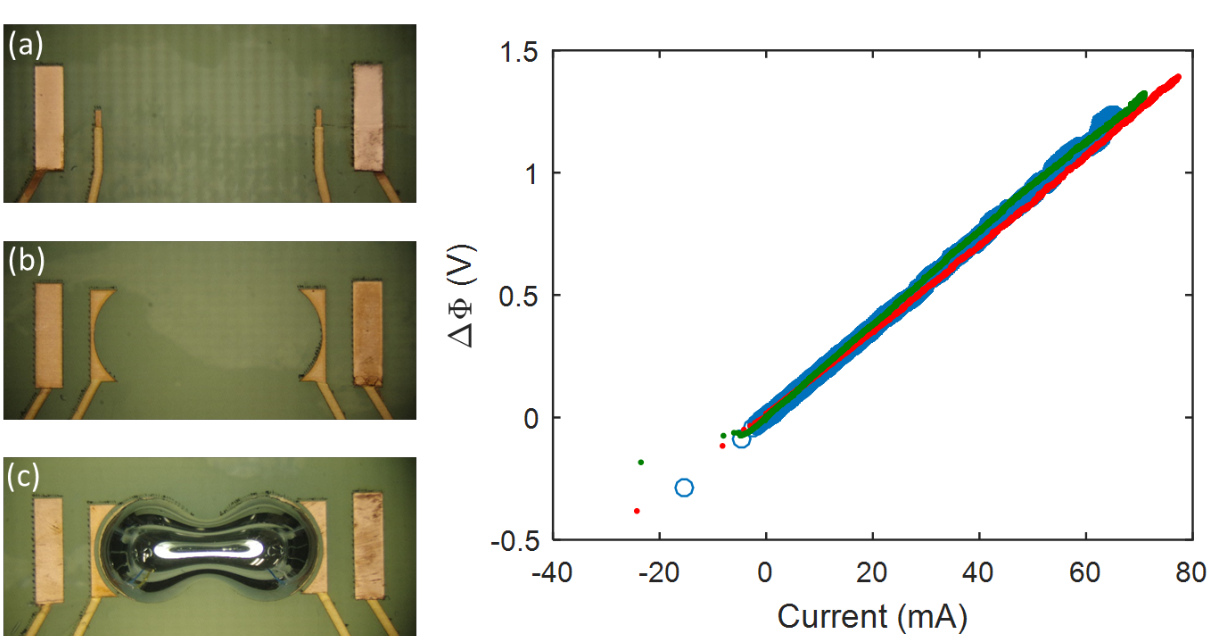

Supplementary Figure 9 – Various testing pads for determining conductivity and their experimental output. (a) Pad design for use with theory (plotted in blue). (b) Arcs for comparison to samples with liquid metal (plotted in red). (c) Arcs with liquid metal to simulate actual experimental conditions (plotted in green).

Although the VES approach provided a good measurement for “effective” conductivity, the finite dimensions of the bath caused inaccuracies with regards to the “true solution” conductivity. The smaller bath resulted in underestimations in solution conductivity, particularly when large outer electrode separations were used. This is due to the fact that the conductivity deeper into the solution (and further from the electrodes) has a larger impact when the electrode separation is increased. When separations are large, the conductivity outside the bath (essentially zero) has a larger influence. The conductivities measured in the small bath (“effective” conductivities) were used for the bipolar electrolysis onset and droplet separation theory in order to remain self-consistent. **The following fits were used in conjunction with experimental conductivity data:**

$$\boldsymbol{Outer\ electrode\ separation}: G_{OES} = -0.0986\,(l_{AB} - 1.5) + 6.5094,$$
$$\boldsymbol{Length\ scale}: G_{SC} = 5.263\,X_{scale}^3 - 19.1416\,X_{scale}^2 + 22.3067\,X_{scale} - 3.6195,$$
$$\boldsymbol{NaOH\ Concentration}: G_C = 4.2069\,C + 0.4475,$$

where G_i is the conductivity (S/m), l_{AB} is the outer electrode separation (mm), X_{scale} is the length scale, and C is the weight per volume concentration of NaOH. No particular constraints were applied during fitting. The length scale curve is nonlinear because bubble production decreases effective conductivity at small scales (bubbles block electrodes) and finite bath size limits effective conductivity at large scales. A max effective conductivity then falls somewhere around a scale of 1.

Measurements were taken in a larger bath (125 mm diameter by 65 mm height, PYREX 3140-125) to gauge the difference in behavior, as seen in Supplementary Figure 10. The results for both small and large baths are compared to values provided by EXW Foxboro, Massachusetts (http://myweb.wit.edu/sandinic/Research/conductivity%20v%20concentration.pdf). The outer electrode separation

plot in Supplementary Figure 11 emphasizes how the small bath results in underestimated conductivity. Even in the larger bath, bubble interference causes an under approximation when the scale is small. In general, however, this method appears to overestimate conductivity, particularly as scale is increased in the large bath. This is likely because the theory assumes point sources while the true pads are rectangular. The average distance from all points on the outer electrodes to the center of the probes (smaller pads of copper) is greater than the distance from center to center. This results in an inflated value for conductivity.

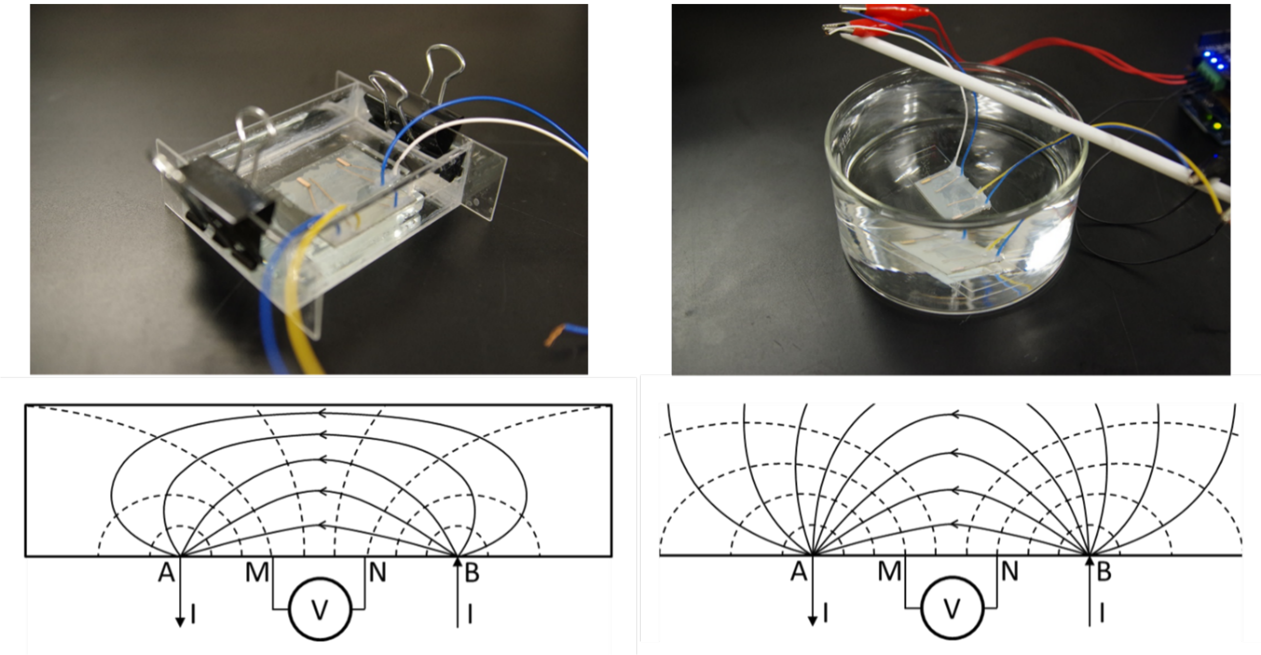

Supplementary Figure 10 – Conductivity tests in various bath sizes. (Left) Small bath. (Right) Large bath. (Below) Approximated current paths (solid lines) and equipotential lines (dotted lines) for each situation.

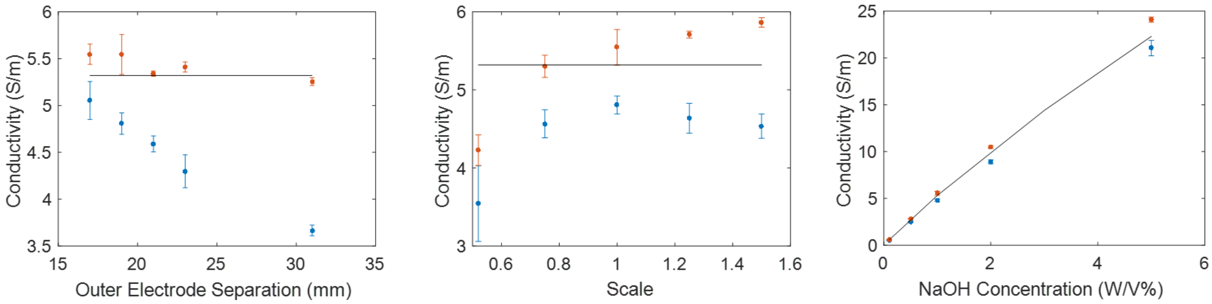

Supplementary Figure 11 – Comparison between conductivity measurements in the small (blue points) and large (red points) baths. Values from literature are reported as a black line. Data is shown for (left) outer electrode separation, (center) scale, and (right) NaOH concentration.

F. Simultaneous Control of Multiple Droplets

SI Video 3 demonstrates how multiple droplets can be controlled in a single bath. Three sets of electrodes are arranged to share a single grounded gate electrode, as shown in Supplementary Figure 12. A set of droplets can be coalesced and separated independently or simultaneously with other pairs of droplets. While the input current for a single pair does influence neighboring liquid metal, it is not sufficient to change the bistable state. Note that, for this demo, there is no electrical connection (ignoring NaOH solution) between the droplets and that they are electrically floating during separation. There is a fundamental limitation to maintaining multiple pairs in a bath – if the pairs are electrically connected, such as linking the source of one pair to the drain of another to create an AND gate, then the inputs to one would influence the other. For example, in the case described above, if the source were anodized to spread and coalesce, the drain of the second pair would also spread and possibly coalesce. Careful arrangements, multiple gate electrodes (rather than a single shared electrode), and isolated baths could overcome many of these challenges.

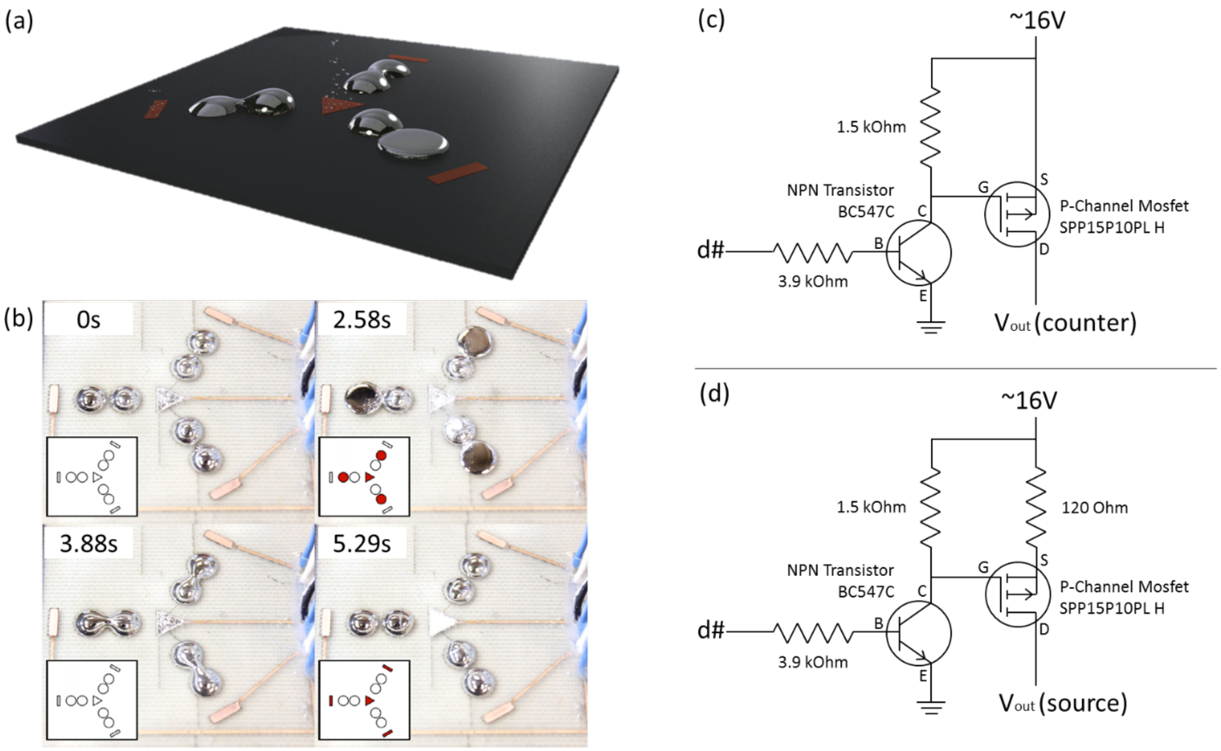

Supplementary Figure 12 – 3 droplet manipulation. (a) Rendering of pad and liquid metal orientation. (b) Sample images from SI Video 3 with timestamps. (c) Circuit for activating counter electrodes for separation. (d) Circuit for activating source electrodes for coalescence.

The circuitry used to control multiple droplets is shown in Supplementary Figure 12c,d. The key components are NPN transistors and P-Channel MOSFETs. The transistors act as level shifters between the Arduino UNO R3 microcontroller (which applies input signals to d#) and the MOSFTETs. As stated above, the central gate electrode was continuously grounded, and the drain electrodes were floating.

Supplementary Figure 12c represents the circuit for controlling a single counter electrode and Supplementary Figure 12d represents the circuit for controlling a single source electrode. The only difference is the addition of a series 120 ohm resistor, which serves to decrease the voltage applied to the source electrode. This is done because spreading and coalescence requires a lesser voltage than separation. The input voltage to the circuit was 16V. This higher voltage enabled the rapid switching demonstrated in SI Video 3.

G. Liquid Metal Transistor

An attractive application for the controlled coalescence and separation of LM is the creation of an electrically-controlled switch. Given the requirements on voltage between source and gate electrodes to achieve spreading and coalescence, we liken this behavior to that of a transistor. The circuit diagram for the liquid metal transistor data is shown in Supplementary Figure 13. The system can be viewed as a high side switch where the source is tied to an input voltage V_{in}, and the drain is then connected to the load – a 10 ohm resistor in addition to a 1 ohm shunt resistor (for current readings) in this example. In the off-state when the droplets are separated, current must pass from the gate LM to the NaOH solution to the drain LM, resulting in a high resistance. When coalesced in the on-state, the drain and source are essentially shorted by the LM bridge.

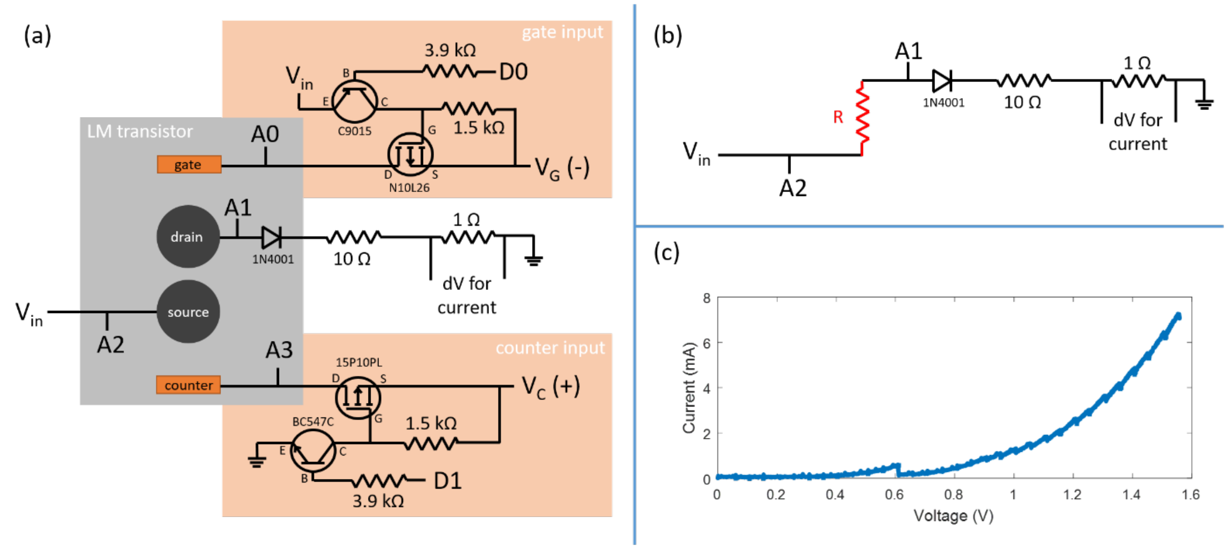

Supplementary Figure 13 – Circuit diagrams and data for liquid metal transistor. (a) Overall setup for controlling the liquid metal transistor, including the gate input, counter input, and the LM transistor, itself. (b) The circuit assumed when calculating the drain-source resistance (as marked in red). (c) The current-voltage curve associated with liquid metal on the source and drain.

The gate input consists of an N-channel MOSFET (N10L26) with an associated PNP transistor (C9015) for level shifting. When activated by a digital signal from an Arduino R3 microcontroller, a negative voltage V_G is applied to the gate electrode of the liquid metal switch. Current flows from the source to the gate, resulting in oxidation, spreading, and droplet coalescence. Similarly, the counter input consists of a P-channel MOSFET (15P10PL) and an NPN transistor (BC547C), allowing the application of a positive voltage V_C to the counter electrode. Current flows from the counter to the source/drain to the gate, causing droplet separation. The diode attached to the drain prevents the unintentional spreading of the LM of that pad during gate activation.

As with the general droplet testing described above, voltages (A0, A1, A2, A3) were monitored with the Extended ADC Shield. Rather than the AD8244BRMZ, the signals were buffered with voltage followers created with quad op amps (LM324M). The current was again tracked with the voltage difference across the 1 ohm resistor by using an instrumentation amplifier (AD623). To simplify the calculation of source-drain resistance, the circuit is simplified to Supplementary Figure 13b. Thus, the results (Supplementary Figure 14) contain features as a result of the signals from gate and counter electrodes.

An important byproduct of the LM-NaOH-LM interface is the role of electrolysis. In particular, if V_{in} is too large, the surface will become more electrochemically active as redox increases. Supplementary Figure 13c captures this behavior is a current-voltage relationship for the drain and source with LM (not including the diode or load). The feature around 0.6V seems to coincide with the onset of significant oxide growth, creating additional resistance and lowering the current, but this aspect requires further study. For the transistor, electrolysis results in a decrease in off-state resistance (higher off-state leakage current). If the input voltage is sufficiently high, bubbles (hydrogen) will form on the drain electrode and the source electrode will grow oxide and begin spreading (see Supplementary Figure 14), possibly causing unintentional coalescence. Given this information, testing focused on input voltages between 0.75V and 1V – high enough to overcome the forward voltage of the diode (~0.7V) and low enough to avoid excessive redox. Note that adjusting the control electronics and replacing the standard diode with a Schottky diode (forward voltage of ~0.3V) could improve the off-state resistance.

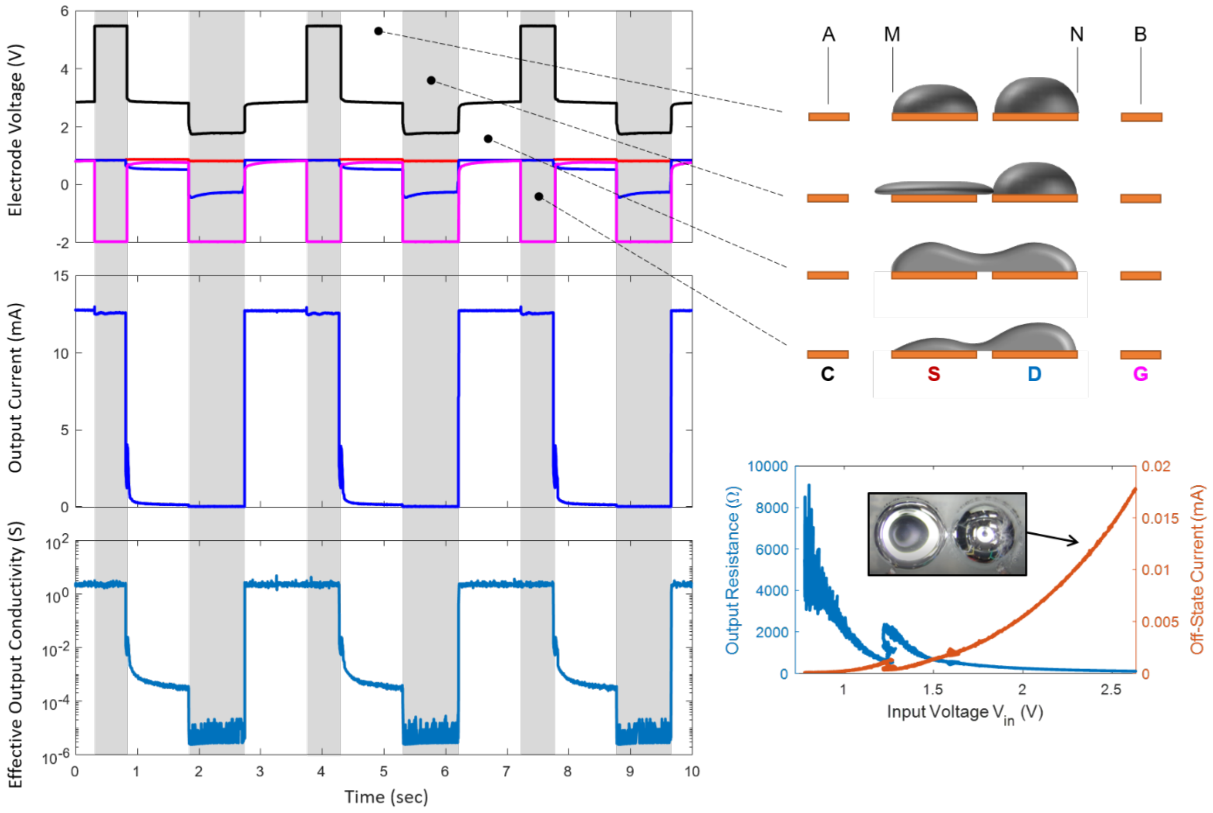

Supplementary Figure 14 – General LM transistor behavior. Left: Plots for electrode voltage, output current, and effective conductivity versus time. Electrode voltages including gate (magenta), counter (black), source (red), and drain (blue). Top right: Droplet states associated with the shaded regions of the plots. States include separated, spreading, coalesced, and separating. Bottom right: Off-state output resistance and off-state leakage current as a function of input voltage. The inset shows bubbling on the drain and unintentional spreading of the source due to electrolysis across these two electrodes.

The transistor behavior can be viewed in SI Video 1. As shown in Supplementary Figure 14, the on-state allows a current of 12.5 mA with a conductivity of ~2 Siemens (less than an Ohm) while the off-state limits current to about 0.1 mA with a conductivity of ~3E-4 Siemens (several kilo-Ohms). The features in current and conductivity are a result of current from the counter/gate during coalescence and separation. During spreading for coalescence, current would flow from drain to gate (negative in this case) if it were not for the diode. As a result, the current drops to nearly zero and the measuring circuitry has difficulty determining a value for resistivity and conductivity. The off-state output resistance and leakage current are also reported. As expected, the resistance falls and the current rises as the input voltage increases and electrolysis becomes more substantial. The feature at ~1.25V is reminiscent of the feature showin in the I-V curve in Supplementary Figure 13. Again, this appears to be caused by the onset of significant oxide growth. The voltage is shifted from ~0.6V to ~1.25V by the forward voltage of the diode.

There is a significant difference between this liquid metal transistor and the theory discussed for the majority of this paper. Bipolar electrodes (and the samples compared to theory in this paper) are floating in the sense that no charge is applied directly to them. However, for this LM transistor, the source and drain are directly tied to V_{in} during droplet separation. During bipolar electrolysis, oxidation and reduction are equal in terms of charge transfer on either side of the electrode – charge in must equal charge out. In this case, however, the direct tie to a voltage source provides an alternative route for charges. Depending on the voltages at the source, gate, and counter, oxidation, reduction, or some ratio of both can occur across the liquid metal. **In other words, tying the source to a voltage input gives control over the level of oxidation and reduction across the liquid metal.** This is why a negative voltage was required for the gate electrode rather than simply using ground as in other experiments. The situation is elucidated by Supplementary Table 1.

For this experiment, the source voltage (V_{in} or V_S) was kept constant as well as the difference between the counter and gate voltages. The potential of the counter and gate were increased progressively while attempting to separate the droplets (held at the source potential V_S) with up to 2 seconds of current flow. When the gate and counter were too high, reduction was excessive and the droplets failed to separate. Likewise, oxidation was excessive and prevented separation when the gate and counter were to low. In some cases (marked "maybe") separation succeeded and failed in the same trial. We can also look at the following ratio: $(V_S - V_G + E_0)/(V_C - V_G)$.), where E_0 is 1.23V for the electrode potential

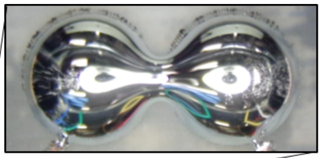

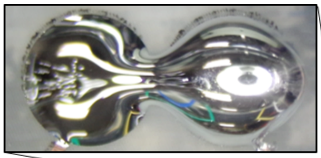

	Trial 1	Trial 2	Trial 3	Trial 4	Trial 5	Trial 6	Trial 7	Trial 8	Trial 9	Floating
**Source(V)**	0.846	0.845	0.845	0.844	0.842	0.842	0.84	0.839	0.836	2.58
**Counter (V)**	5.81	5.76	5.71	5.66	5.55	5.51	5.47	5.43	5.33	7.44
**Gate (V)**	-1.62	-1.67	-1.73	-1.78	-1.83	-1.88	-1.93	-1.97	-2.07	0
**Counter-Gate (V)**	7.43	7.43	7.44	7.44	7.38	7.39	7.4	7.4	7.4	7.44
**Ratio**	0.497	0.504	0.511	0.518	0.529	0.535	0.541	0.546	0.559	0.512
**Separation?**	No	No	Maybe	Yes	Yes	Maybe	Maybe	No	No	Yes

Supplementary Table 1 – Table for adjusting the ratio between source, counter, and gate during droplet separation. The images above represent separation attempts with excess reduction (left) and excess oxidation (left).

difference between the copper and GaIn. There is a narrow region from approximately 0.511 to 0.541 where separation is possible a range of about 0.2V in this case. It turns out that the ratio for the floating electrode case falls on the lower end of the range, around 0.512. The implication is that the bipolar electrolysis provides an approximately optimal level of oxidation and reduction for droplet separation. Furthermore, the theories presented in this paper are still applicable for this liquid metal transistor. As a side note, it is not known why the ratio does not fall exactly on 0.5 for equal oxidation and reduction. Possible reasons include voltage divider effects due to oxide growth and bubbles or changing electrode potentials as oxidation occurs.

H. Progressive Decrease of Voltage for Separation

This experiment was implemented with the same circuitry and methods as the general testing described above. However, instead of increasing the voltage during separation, the ~9V was immediately applied and decreased by 0.1V per second. Of particular interest were the larger scales, such as x1.25, where successful separation only occurred in 11 out of 20 attempts during parametric testing. The plots shown in Supplementary Figure 15 are for a x1.25 sample. In the first 9 trials, no separation occurred. However, from trial 10 onwards, the LM droplets successfully separated. During the 14th trial, separation occurred with a counter electrode voltage of 6.72V and a current of 68.0 mA. On the 15th trial, the test process was reverted to the original method of increasing voltage until separation. Interestingly, separation occurred at a voltage of 6.85 and a current of 69.5 mA. The proximity of the values between these two trials indicates that there is a narrow region in which separation occurs. It is unclear why an upper limit exists, though it could be a result of countering surface tension effects from continuous

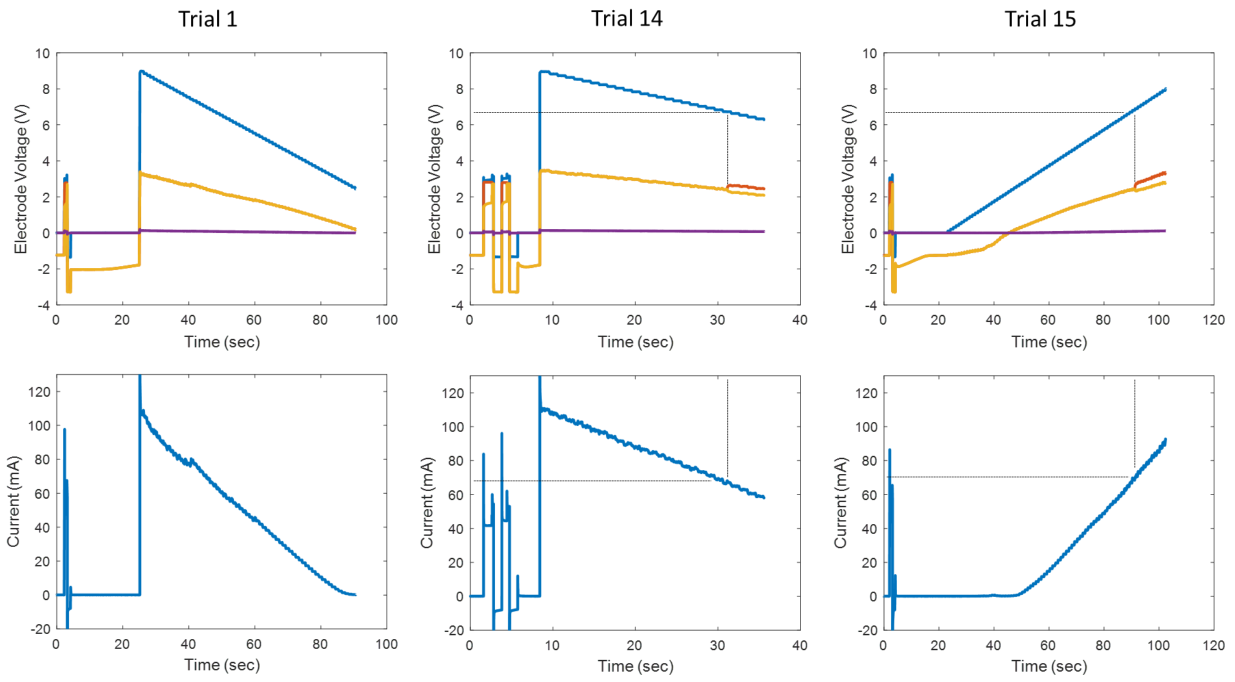

Supplementary Figure 15 – Plots for voltage (blue – counter, red – source, yellow – drain, purple – gate) and applied current of a x1.25 scale sample. Left column: The 1st trial. Middle column: The 14th trial. Right column: The 15th trial, increasing voltage and current instead of decreasing.

electrowetting.

This data also suggests that the solution, electrodes, or liquid metal are somehow being altered during testing. While this was kept to a minimum during experimentation by limiting testing cycles, using fresh NaOH solution, and testing on multiple PCB electrodes, it is an aspect that needs further exploration. It should be noted that after excessive testing, copper electrodes are consumed, likely the result of copper oxidation followed by corrosion by the NaOH solution. Inert platinum or gold electrodes would be preferential for future experiments and prototyping.

The same experiment was performed on a scale x1 sample. The very first sample separated with a voltage of 8.02V and a current of 73.0 mA. By the third trial, separation occurred immediately at 9.41V and 90.4 mA. Note that separation on the upward ramp generally required about 50.9 mA for separation. We conclude that increasing the scale increases the lower limit for separation and decreases the upper limit. At a scale of x1.5, there is no window for successful switching. The reason for this behavior is unclear, but the fact that scaling down the device does not suffer from this limitation is promising.

It should be noted that, as shown in Supplementary Figure 16, initial simulations with Surface Evolver indicate that increasing the scale increases the interfacial tension gradient required for separation. At larger scales, the dominance of gravitational forces prevent separation as the droplets simply flatten under their own weight. In fact, separation failed in the simulations at x1.5 due to excessive spreading which led to an instability. At smaller scales, the gravitational forces vanish, and the required gradient appears to level out.

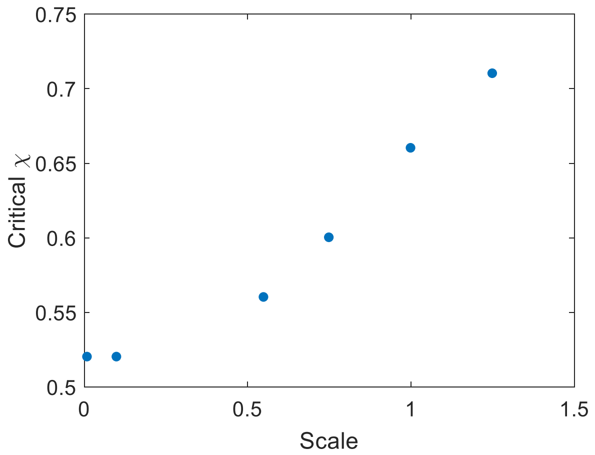

Supplementary Figure 16 – Surface Evolver results for scale verses interfacial tension gradient, χ, required for separation.

I. Droplet Separation Power Requirements

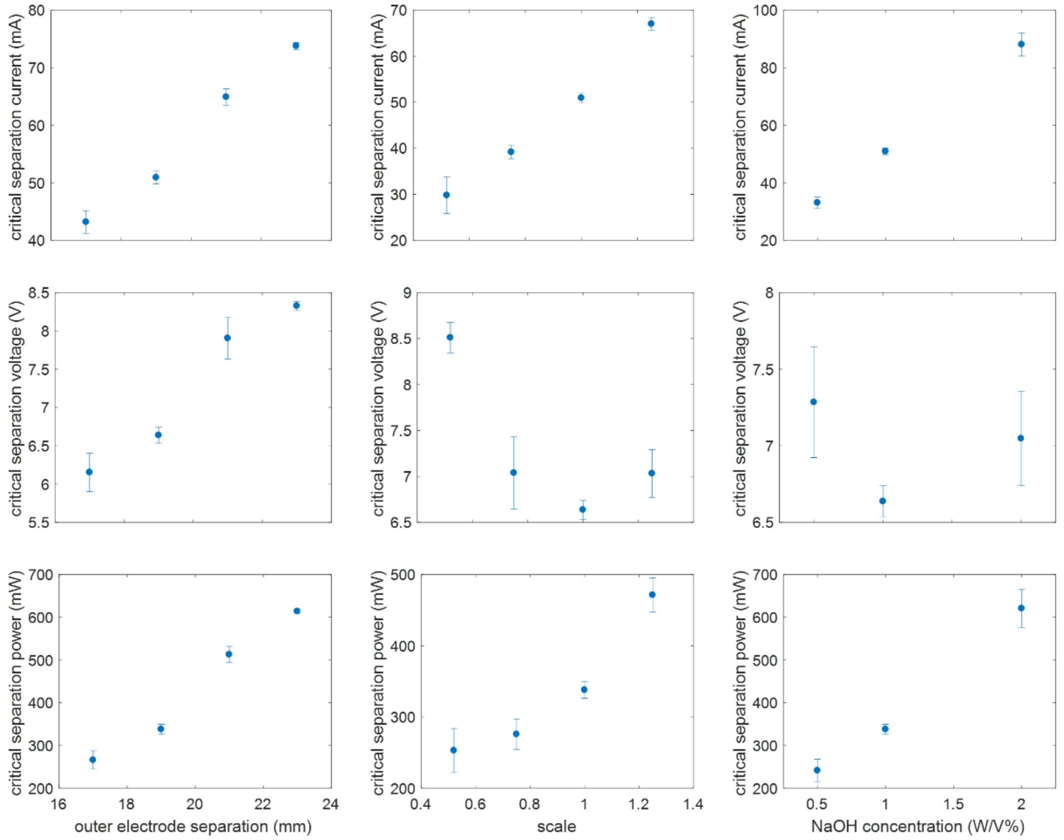

Supplementary Figure 17 – Data for voltage, current, and power requirements for droplet separation under various outer electrode separations, length scales, and NaOH concentrations.

Of particular interest in Supplementary Figure 17 are the power requirements. A number of approaches can be taken to improve efficiency. According to experimental results, decreasing outer electrode separation drives down both voltage and current. Essentially, current has to flow through less solution as gaps are decreased. However, as electrodes are placed in closer proximity, interference from bubbling and turbulence at the outer electrodes could become an obstacle. Scale has a similar effect on power. Interestingly, voltage requirements remain nearly constant until a scale of 0.5. The constant voltage requirements is a result of the electric field scaling approximately inversely with distance (distance decreases, electric field increases, and voltages stay constant). However, bubble formation drastically increases resistance and voltage requirements at small scales. Finally, decreased NaOH concentration decreases power, as well. Again, voltage requirements remain fairly constant, this time due to the constant dimensions. The drawback here is that lower concentrations leads to slower overall functionality (both separation and coalescence). At 0.1%, separation failed altogether due to apparent lack of ionic species (lack of conductivity).

J. Supplementary Information Citations

[1] Tate, T. On the magnitude of a drop of liquid formed under different circumstances. *London, Edinburgh, Dublin Philos. Mag. J. Sci.* **27,** 176–180 (1864).

[2] Zhang, Z. & Mori, Y. H. Formulation of the Harkins-Brown correction factor for drop-volume description. *Ind. Eng. Chem. Res.* **32,** 2950–2952 (1993).

[3] Garandet, J. P., Vinet, B. & Gros, P. Considerations on the Pendant Drop Method: A New Look at Tate's Law and Harkins' Correction Factor. *J. Colloid Interface Sci.* **165,** 351–354 (1994).

[4] Huttenlocher, D. P., Klanderman, G. A. & Rucklidge, W. J. Comparing images using the Hausdorff distance. *IEEE Trans. Pattern Anal. Mach. Intell.* **15,** 850–863 (1993).

[5] Rieger, P. H. in *Electrochemistry* 109–150 (Chapman & Hall, Inc, 1994).

[6] Wang, M., Wang, Q. & Karki, B. Arts of electrical impedance tomographic sensing. *Philos. Trans. R. Soc. A Math. Phys. Eng. Sci.* **374,** 20150329 (2016).

[7] Mavré, F. *et al.* Bipolar Electrodes: A Useful Tool for Concentration, Separation, and Detection of Analytes in Microelectrochemical Systems. *Anal. Chem.* **82,** 8766–8774 (2010).
